# Intravital imaging in spontaneously hypertensive stroke-prone rats-a pilot study

**DOI:** 10.1186/2040-7378-6-1

**Published:** 2014-01-25

**Authors:** Solveig Niklass, Stoyan Stoyanov, Cornelia Garz, Celine Z Bueche, Stine Mencl, Klaus Reymann, Hans-Jochen Heinze, Roxana O Carare, Christoph Kleinschnitz, Stefanie Schreiber

**Affiliations:** 1 Department of Neurology, Otto-von-Guericke-University, Leipziger Strasse 44, 39120 Magdeburg, Germany; 2 German Center for Neurodegenerative Diseases (DZNE), Magdeburg, Germany; 3 Department of Neurology, University Hospital of Würzburg, Würzburg, Germany; 4 Leibniz Institute for Neurobiology (LIN), Magdeburg, Germany; 5 Faculty of Medicine, University of Southampton, Southampton, UK

**Keywords:** SHRSP, Intravital imaging, 2 PM, CSVD

## Abstract

**Background:**

There is growing evidence that endothelial failure and subsequent blood brain barrier (BBB) breakdown initiate cerebral small vessel disease (CSVD) pathology. In spontaneously hypertensive stroke-prone rats (SHRSP) endothelial damage is indicated by intraluminal accumulations of erythrocytes (erythrocyte thrombi) that are not observed with current magnetic resonance imaging techniques. Two-photon microscopy (2 PM) offers the potential for real-time direct detection of the small vasculature. Thus, within this pilot study we investigated the sensitivity of 2 PM to detect erythrocyte thrombi expressing initiating CSVD phenomena in vivo.

**Methods:**

Eight SHRSP and 13 Wistar controls were used for in vivo imaging and subsequent histology with haematoxylin-eosin (HE). For 2 PM, cerebral blood vessels were labeled by fluorescent Dextran (70 kDa) applied intraorbitally. The correlation between vascular erythrocyte thrombi observed by 2 PM and HE-staining was assessed. Artificial surgical damage and parenchymal Dextran distribution were analyzed postmortem.

**Results:**

Dextran was distributed within the small vessel walls and co-localized with IgG.

Artificial surgical damage was comparable between SHRSP and Wistar controls and mainly affected the small vasculature. In fewer than 20% of animals there was correlation between erythrocyte thrombi as observed with 2 PM and histologically with HE.

**Conclusions:**

Contrary to our initial expectations, there was little agreement between intravital 2 PM imaging and histology for the detection of erythrocyte thrombi. Two-photon microscopy is a valuable technique that complements but does not replace the value of conventional histology.

## Introduction

Human cerebral small vessel disease (CSVD) is commonly found in the brains of the elderly and in Alzheimer’s disease (AD) as demonstrated in autopsy and human imaging studies [1-8]. CSVD affects the capillaries and small arteries and is initiated by an early endothelial damage with subsequent blood brain barrier (BBB) breakdown [9-15]. Current human magnetic resonance imaging (MRI) techniques fail to directly detect the initial stages of the pathological process and instead identify advanced CSVD features including white matter hyperintensities (WMH), microbleeds, recent subcortical infarcts and lacunes already associated with cognitive decline [11,16-18]. The development of efficient prophylactic and early therapeutic strategies for CSVD requires the establishment of biomarkers indicative of the early endothelial failure that are identifiable by imaging.

Spontaneously hypertensive stroke-prone rats (SHRSP) develop all features of human CSVD including an endothelial dysfunction with resulting BBB damage indicated by plasma protein leakage into the small vessel walls and the perivascular parenchyma [13]. Subsequent small vessel wall thickening occurs and increasing fragility of the vasculature leads to perivascular bleeds, reactive microthromboses and associated tissue infarcts [12,19].

The early endothelial damage in SHRSP results in an activated coagulatory state followed by the formation of an intravasal mesh of thrombocytes and the von-Willebrand-factor (vWF) [14]. Within that mesh, erythrocytes adhere and aggregate and, thus, become visible as erythrocyte thrombi by the conventional histology [12]. Therefore, erythrocyte thrombi could serve as the sought biomarker indicative of initiating CSVD pathologies. Indeed, first pilot 3Tesla (T) and 4.7 T MRI studies failed to visualize those erythrocyte thrombi; more recently, high field MRI experiments have been undertaken [12,20].

The main advantages of two-photon microscopy (2 PM), an additional intravital imaging technique, include its potential for the direct visualization of the small, cortical, vasculature including capillaries and arterioles, with the disadvantage associated with the invasive nature of the technique [21-23]. Intravital microscopy is performed using fluorescent labeled Dextran to visualize cerebral vessels and thus, blood flow can be determined by velocity measurements of erythrocytes, which are directly visible within the small vasculature [22-29].

The 2 PM has been established in different rat models including Wistar and Sprague Dawley rats without intrinsic cerebrovascular pathologies [23,30,31] and spontaneously hypertensive rats (SHR), an animal model of chronic arterial hypertension with a rather low incidence of spontaneous infarct development [13,32]. SHR and SHRSP both develop arterial hypertension and an associated vascular pathology; however, the pathology is more severe in SHRSP [33]. Within this first pilot study we aimed to investigate whether 2 PM could be performed safely in SHRSP, an appropriate model of CSVD pathology, with consecutive high spontaneous infarct frequencies. We analyzed the vulnerability to artificial surgical damage. Furthermore, we investigated the sensitivity of 2 PM to detect erythrocyte thrombi. Our hypothesis was that intravital 2 PM is suitable for the direct visualization of intravasal erythrocyte clusters. Combining MRI and 2 PM approaches could therefore provide new insights into the establishment of biomarkers indicative of early CSVD pathology.

## Materials and methods

### Animals

All experiments were approved by the local Animal Care Committee of Saxony-Anhalt (42502-2-1148 DZNE). Fifteen male SHRSP (Charles River Laboratories International Inc., Wilmington, MA, USA) aged from 30 to 32 weeks (w) and 15 male Wistar rats (Charles River Laboratories International Inc., Wilmington, MA, USA) at different age groups (17-21 w - n = 4, 27-29 w - n = 3, 30-32 w - n = 4, 33-36 w - n = 4) were investigated. Eight SHRSP were used for in vivo two-photon imaging and 7 SHRSP underwent sole in vivo Dextran application without intravital imaging to investigate the Dextran distribution within the tissue. Age groups were chosen because of the high likelihood to detect erythrocyte thrombi in 30 to 32 w old SHRSP. As previously demonstrated, we expected the young Wistar controls to be free of erythrocyte thrombi and therefore serving as an excellent control group [12]. All animals were housed with a natural light-dark cycle and allowed to access water and food *ad libitum*.

### Two-photon imaging

#### A. Anesthesia and cranial window preparation

All rats were anesthetized by intraperitoneal injections of Pentobarbital (1 mL per 100 g body weight); depth of anesthesia was tested by the absence of reflections after a painful stimulus. During surgery the body temperature was maintained at 37°C with a thermostatically controlled warming pad (Harvard Apparatus, March, GER). After shaving the skull and its fixation in a stereotactic frame (Stoelting Europe, Dublin, IRL), the scalp was lifted with a forceps (Fine Science Tools (FST), Heidelberg, GER) and opened by surgical scissors (FST, Heidelberg, GER). Within the surgical area the periosteum was removed from the surface of the skull with a sharp double-spoon (sharp spoon after Willinger; Intermedical24, Regensburg, GER) over the surgical area. Bleeding vessels were closed with a cautery kit (Bovie Medical, Clearwater, USA) and the skull was cleaned and disinfected with a 3% H_2_O_2_ solution (Carl Roth, Karlsruhe, GER). Under a surgical microscope (Carl Zeiss Meditec, Oberkochen, GER) the skull was thinned with a Dremel (Dremel Europe, Konijnenberg NL) and a drill (Eickemeyer, Tuttlingen, GER) was used to prepare the cranial window over the parietal cortex located between -2 mm and -6 mm in relation to the Bregma and 1 mm to 5 mm lateral in relation to the sagittal suture. The skull was regularly cleaned with compressed air (CRC Industries UK Ltd, Bridgwater, UK) to avoid soiling of the field in which surgery was conducted. After removing the bone piece carefully, a small syringe needle (B. Braun, Melsungen, GER) was used to make a little incision and remove the dura mater to the edge of the cranial window by using a spring scissor (FST, Heidelberg, GER). In mice, intravital imaging is usually performed throughout the intact dura mater [29], thus, an additional preparation without removing the dura mater was attempted in two of the 15 Wistar rats, too. After surgery the cranial window was filled with sterile irrigation (e.g. sodium chloride 0,9%; B. Braun, Melsungen, GER) and sealed by a 7 mm cover glass (Gerhard Menzel, Braunschweig, GER) using the instant glue Roti-coll 1 (Carl Roth, Karlsruhe, GER).

#### B. In vivo imaging

After surgery, in vivo imaging was performed with the LSM 7 MP multiphoton microscope (Carl Zeiss Microscopy, Oberkochen, GER) using the Chameleon Vision 2 laser (Coherent Inc., Santa Clara, CA, USA). To image cerebral blood vessels, the blood plasma was marked by the fluorescently labeled dye Dextran (70 kDa, 10 mg/mL, Life Technologies, Darmstadt, GER), applied intraorbitally with a small syringe (B. Braun, Melsungen, GER). For imaging, the skull of the rats was fixed in a head holder (Luigs und Neumann, Ratingen, GER) and the animals were placed on a heating plate (Physitemp Instruments, Clifton, NJ, USA) under the microscope for one imaging session. The known maximum achievable depth for in vivo imaging in rats is up to 500 μm [34,35], confirmed by our measurements covering all cortical layers with maximal imaging depths of 300 μm to 500 μm.

### Perfusion

All animals were transcardially perfused directly after imaging with 120 mL phosphate-buffered saline (PBS) followed by fixation through perfusion with 120 mL of 4% paraformaldehyde (PFA) within 8 minutes. After decapitation, the brains were removed, fixed in 4% PFA for 48 hours, placed into 30% sucrose for cryo-protection for 6 days, and frozen in methylbutane (Carl Roth, Karlsruhe, GER) at -80°C.

### Histology

Twenty-four hours before coronal slices (30 μm) were prepared the brains were stored at-20°C. The whole brains of all animals were sliced from the frontal to the occipital pole using a cryotoma (Leica Biosystems, Nussloch, GER). Per animal there were 10 sectional planes with a distance of 930 μm between the frontal pole and the first sectional plane and a distance of 1 mm between each sectional plane respectively. Of each sectional plane three slices per brain and therefore 30 slices per rat were stained with Haematoxylin-Eosin (HE). The surgical area was located between the 5^th^ and the 9^th^ sectional plane, thus, the distances between the frontal pole and the surgical area were about 5 mm to 9 mm respectively and thus, the surgical area covered about 4 mm of the brain. Of the 5 sectional planes covering the surgical area, 15 slices per animal were available for histological analysis. In brief, HE-staining was performed as follows: slices were washed with distilled water, immersed in Haemalaun solution after Mayer (Carl Roth, Karlsruhe, GER) for 5 minutes, washed again, blued under running tap water for 10 minutes and stained with Eosin (Carl Roth, Karlsruhe, GER) for 40 seconds. Dehydration of the slices by immersion in increasing concentrations of alcohol (Rotisol; Carl Roth, Karlsruhe, GER) and in Xylene (Carl Roth, Karlsruhe, GER) was followed by mounting with Histomount (Shandon Histomount™ Xylene Substitute Mountant; Fisher Scientific, Schwerte, GER).

### Immunohistochemistry

Brain slices of 9 SHRSP (3 with 2 PM, 6 with sole Dextran application without intravital imaging, 4-5 slices per animal) and 1 Wistar rat (with 2 PM, 4 slices) were investigated immunohistochemically. Slices were adjacent to the slices used for HE-staining. In short, repeated washing of the slices in PBS and blocking with 0.1 mol/L PBS, 0.5% Triton-X (Carl Roth, Karlsruhe, GER) and 10% donkey serum (Sigma-Aldrich, St Louis, MO, USA) was followed by immunohistochemical staining with solanum tuberosum lectin-fluorescein isothiocyanate (STL-FITC, endothelial marker [36], 1:500; Axxora, Enzo Life Sciences GmBH, Lörrach, GER) overnight at 4°C in PBS containing 5% donkey serum. Slices were washed anew before application of Cy5-donkey anti-rat Immunglobulin G (IgG, 1:200; Jackson ImmunoResearch, West Grove, PA, USA) for 2 hours at room temperature. Cy5-donkey anti-rat IgG was used for detection of BBB breakdown [36]. Finally DAPI staining (DAPI = 4′.6-Diamidin-2-phenylindol, 1:10000; MoBiTec, Göttingen, GER) was performed for 20 minutes at room temperature. In vivo applied Dextran (Dextran, Tetramethylrhodamine; Life Technologies, Darmstadt, GER) was visualized post mortem by Tetramethylrhodamine labeling. After dehydration with increasing concentrations of alcohol, slices were mounted on slides with Histomount.

### Quantification and statistics

The surgical area was investigated histologically in all SHRSP and Wistar control rats to detect artificial damage (infarcts, hemorrhages, small bleeds, partial thromboses) caused by surgery. Additionally, all capillaries and arterioles in the surgical area were examined for the occurrence of erythrocyte thrombi within 2 PM and HE-stained slices. Quantification was performed in a binary manner (existent, not existent), respectively. For all parameters (artificial damage, erythrocyte thrombi) student’s t-test was used to calculate the differences between the controls and SHRSP group. P-values ≤0.05 were deemed to be statistically significant.

## Results

### Surgery and imaging

**Figure 1:**
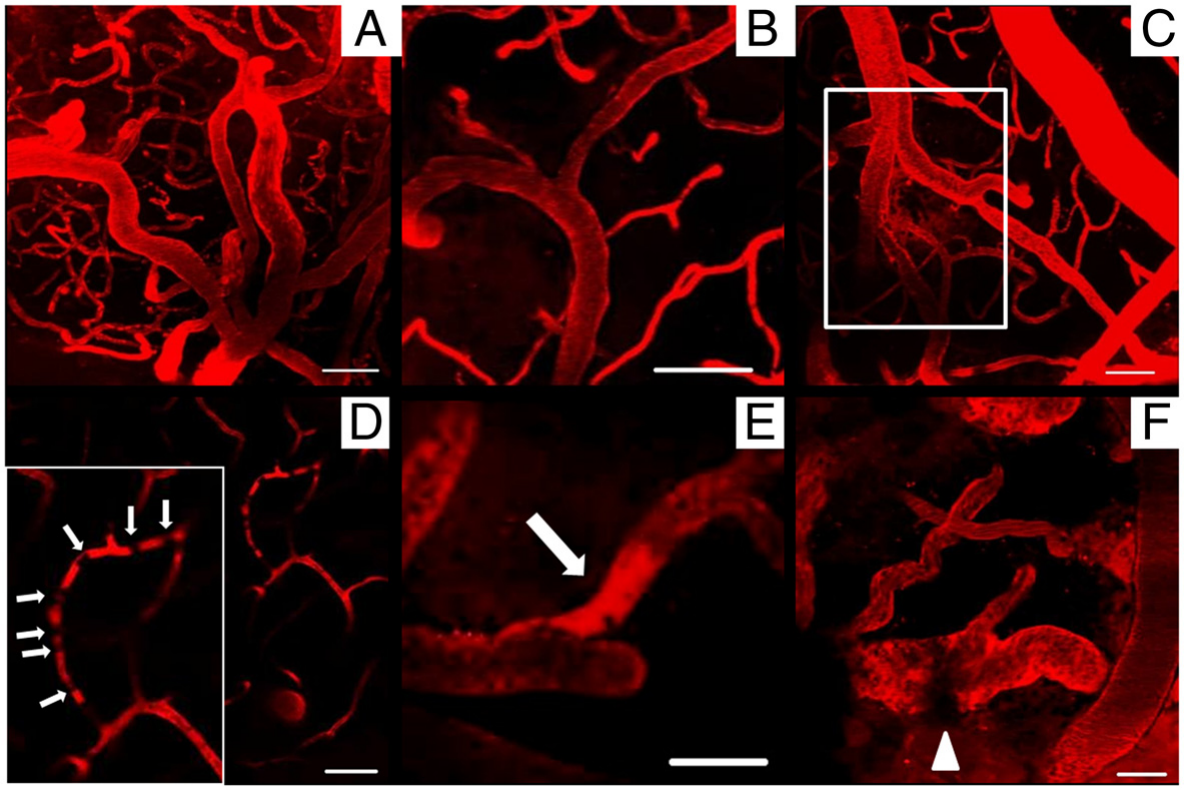
**Two-photon microscopy of small cortical vessels.** In vivo imaging of SHRSP and Wistar control rats revealed the excellent detectability of arterioles and the capillary bed (**A**, SHRSP, 30 w). Non pathological vessels (**B**, Wistar, 31 w) and arteriolar Dextran leakage indicative for vessel wall damage with suspected perivascular bleeds (**C**, SHRSP, 31 w) were detected. **D** (SHRSP, 31 w) shows a capillary with suspected erythrocyte thrombi indicated by not moving dark spots (local gaps of Dextran contrast agent, white arrows), **E** (SHRSP, 30 w) displays the accumulation of Dextran in an arteriole (white arrow). The arrowhead in **F** (Wistar, 36 w) marks a rather plane lack of contrast agent in the vessel wall possibly indicative of endothelial injuries. w – age in weeks, scale bars = 50 μm.

Surgery and anesthesia were well tolerated by both rat groups. None of the animals died during the imaging sessions. In two control animals imaging was not successful so they were not investigated histologically. The cerebral blood vessels, including small arteries, arterioles and the capillary bed, could be visualized well and reproducibly by 2 PM (Figure 1A). In addition to vessels without pathological features (Figure 1B), leakage of the contrast agent out of the small vessel walls histologically confirmed as bleeding (Figure 1C) was detected and black dotted intravasal and wall adherent contrast agent gaps were suspected erythrocyte thrombi (Figure 1D and E). Additional features of erythrocyte thrombi suspected in the 2 PM included no or slow movements of the “contrast agent gaps”. Rather plane lacks of contrast agent in the small vessel wall (Figure 1F, white arrowhead) might be interpreted as endothelial injury.

In the two Wistar rats without dura mater removal, predominantly the blood vessels of the intact dura mater were visible. The depth for imaging of cortical vessels was thus restricted to about 70 μm (1^st^ cortical layer [37]) instead of about 500 μm (3^rd^ cortical layer [37]) in the remaining animals with dura mater removal. Moreover in both Wistar rats without dura mater removal the blood vessels of the dura mater blasted through repeated laser illumination, so no further recording was possible.

### Artificial vascular damage

**Figure 2:**
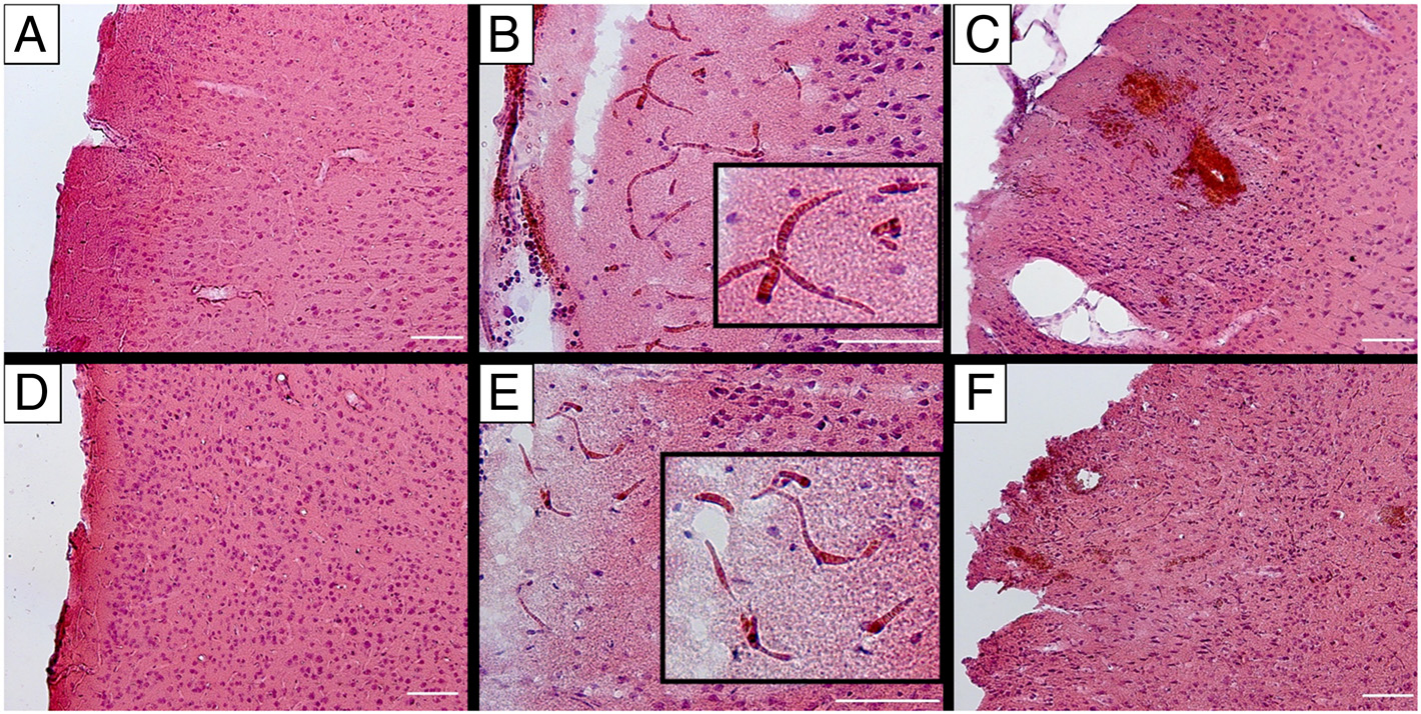
**HE-staining of the surgical area in SHRSP and controls with dura removal.** The surgical area of SHRSP (**A**, 30-32 w) and Wistar control rats (**D**, 30-32 w) and artificial damage in both groups (**B** and **C**, SHRSP; **E** and **F**, Wistar controls) cannot be distinguished. Both, SHRSP **(A)** and Wistar **(D)** show surgical fields that are free of any artificial vascular damage, but also areas with partial capillary thromboses (**B**, SHRSP; **E**, Wistar control) and small artificial bleeds (**C**, SHRSP; **F**, Wistar control). w – age in weeks, scale bars = 100 μm.

**Figure 3:**
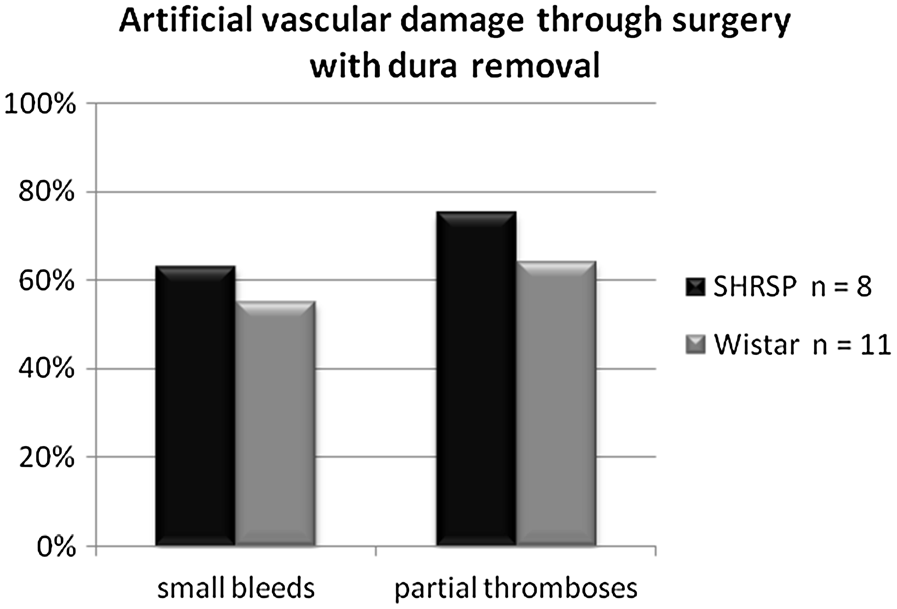
**Histogram of artificial vascular damage through surgery with dura removal.** Prevalence of artificial vascular damage through surgery including partial capillary thromboses and small bleeds was comparable between SHRSP and Wistar control rats.

SHRSP (Figure 2A-C) and Wistar control rats (Figure 2D-F) showed no histological differences in the surgical area (Figure 3). In both groups there were surgical fields without any artificial vascular damage (Figure 2A,D). Artificial damage in the surgery area included the detection of densely packed erythrocytes within capillaries, indicating partial thrombus formation (partial thromboses, Figure 2B,E) and small bleeds (Figure 2C,F). Thrombi likely hampered the Dextran to flow through the plugged capillaries and thus, were not visible with 2 PM imaging. Contrary, “normal” capillary erythrocyte thrombi indicative of endothelial and BBB failure (initiating phenomena of CSVD pathology in SHRSP, [12]) were characterized by intravasal erythrocytes which were commonly well distinguishable from each other and regularly preserving their shapes (see [12,14]). Thus, erythrocyte thrombi and artificial partial thromboses could be clearly distinguished. Both artificial phenomena, partial capillary thromboses and small bleeds, occurred with equal frequencies within the SHRSP and the control group (Figure 3). Severity of artificial damage in terms of the frequency of capillary thromboses and small bleeds per affected animal did not differ between the groups (thromboses: p = 0.078; small bleeds: p = 0.206). None of the rats developed any tissue infarctions or large parenchymal hemorrhages within the surgical field.

### Detection of intravasal erythrocyte thrombi

In conventional histology performed after intravital imaging, the prevalence of erythrocyte accumulations in terms of erythrocyte thrombi was 100% within the surgical field of SHRSP (Table 1). Thereby, the capillary bed was affected in total (100%), whereas the arterioles in only three (38%) SHRSP (Table 1). In contrast, fewer Wistar control animals exhibited erythrocyte thrombi within the capillaries (n = 3, 23%) and arterioles (n = 3, 23%, Table 2). Histological prevalence of intravasal erythrocyte thrombi was significantly higher in the SHRSP group compared to the controls (p = 0.005), a result in line with our previous work [12]. Contrary, according to our definitions of intravital erythrocyte thrombi appearance (see Results, part Surgery and Imaging) 2 PM assumed capillary erythrocyte thrombi in only two SHRSP (13%, Table 1) and two Wistar rats (15%, Table 2), and arteriolar erythrocyte thrombi in three SHRSP (38%, Table 1) and none of the controls (Table 2).

**Table 1:** Erythrocyte thrombi (stases) in the surgical field of SHRSP suspected by 2 PM and confirmed histologically

**SHRSP**	**HE-staining (confirmed)**		**2 PM (suspected)**	
**Age animals**	**Capillary stases**	**Arteriolar stases**	**Capillary stases**	**Arteriolar stases**
**30 w** 701	1	1	0	0
711	1	0	0	1
**31 w** 623	1	1	0	0
712*	1	1	1	1
713	1	0	0	0
714	1	0	0	1
**32 w** 620	1	0	0	0
621	1	0	0	0
Total number	8	3	1	3
in%	100%	38%	13%	38%

HE-investigations of SHRSP revealed the affection of all animals (100%) by capillary erythrocyte thrombi, whereas 2 PM failed to confirm those capillary erythrocyte aggregations in most of the rats. Only three SHRSP showed arteriolar erythrocyte thrombi that could be detected intravitally in only one of those rats (SHRSP 712). Only in SHRSP 712 (asterisk) imaging and histological data were matched well. Within the remaining SHRSP results obtained from histology and intravital microscopy completely differed.

1 – existent, 0 – not existent.

**Table 2:** Erythrocyte thrombi (stases) in the surgical field of Wistar rats suspected by 2 PM and confirmed histologically

**Wistar**	**HE-staining (confirmed)**		**2 PM (suspected)**	
**Age animals**	**Capillary stases**	**Arteriolar stases**	**Capillary stases**	**Arteriolar stases**
**17 w** 47	0	0	0	0
**20 w** 48	0	0	0	0
49	0	0	0	0
**27 w** 52**	0	0	0	0
**29 w** 55**	0	0	0	0
**30 w** 89	0	0	0	0
**31 w** 60	1	1	0	0
90	0	0	1	0
**32 w** 57	0	0	1	0
**33 w** 62	0	0	0	0
**34 w** 59	0	1	0	0
**35 w** 64	1	0	0	0
**36 w** 65	1	1	0	0
Total number	3	3	2	0
in%	23%	23%	15%	0%

HE-investigations of Wistar control rats revealed erythrocyte thrombi within the capillaries (n = 3 animals, 23%) and arterioles (n = 3 animals, 23%). With intravital imaging in some animals capillary (n = 2, 15%) but no arteriolar erythrocyte accumulations were suspected. Overall, results obtained from both techniques did not fit in any of the controls.

1 – existent, 0 – not existent; ** – animals without dura mater removal.

**Figure 4:**
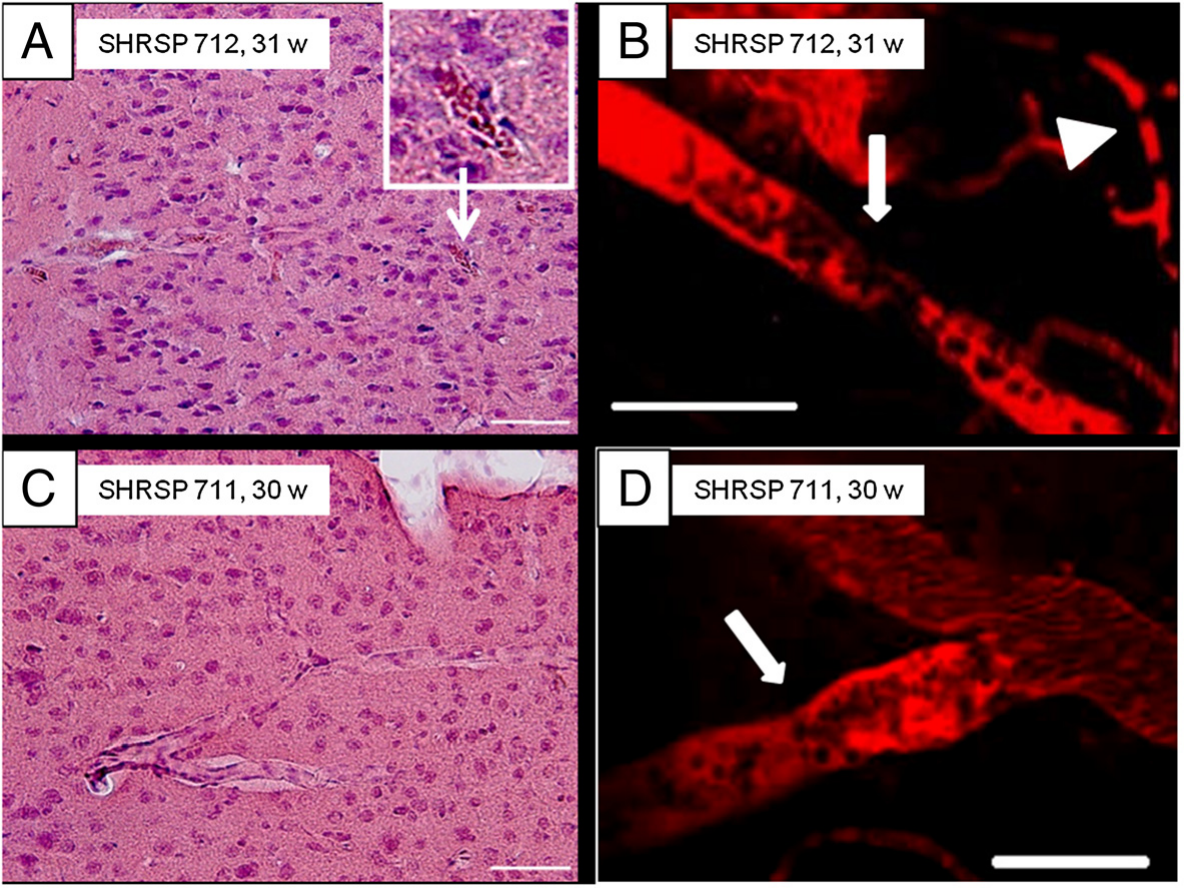
**Detection of intravasal erythrocyte thrombi.** In vivo imaging was performed before brain slices were investigated by HE-staining. Comparison of the results independently revealed from both techniques showed quite well matched data in only one animal (SHRSP 712, Table 1). Here, the intravitally suspected arteriolar (**B**, white arrow) and capillary (**B**, white arrowhead) erythrocyte thrombi match those found histologically (**A**, inlay shows an exemplary arteriolar erythrocyte thrombus). In contrast, data of SHRSP 711 (Table 1) represent exemplary results indicative of the mismatch between imaging and histological data found in the remaining animals. Here, arteriolar erythrocyte thrombi were suspected with 2 PM (**D**, white arrow), but no arteriolar erythrocyte thrombi were found within the whole surgical area **(C)**. w – age in weeks, scale bars = 50 μm.

The occurrence of erythrocyte thrombi suspected with intravital imaging and confirmed histologically was thus only 17% for arteriolar erythrocyte thrombi (in 1 out of 6 animals, Tables 1 and 2) and even lower for capillary erythrocyte thrombi (9%, in 1 out of 11 animals, Tables 1 and 2). For example, images from animal SHRSP 712, the only animal with histological and in vivo similar set of data in agreement with each other (Table 1, asterisk), are shown in Figure 4A and B. Data of SHRSP 711 as an example demonstrate the non-concordance of imaging and histological results regarding the detection of erythrocyte thrombi (Figure 4C and D).

### Dispersal of dextran

**Figure 5:**
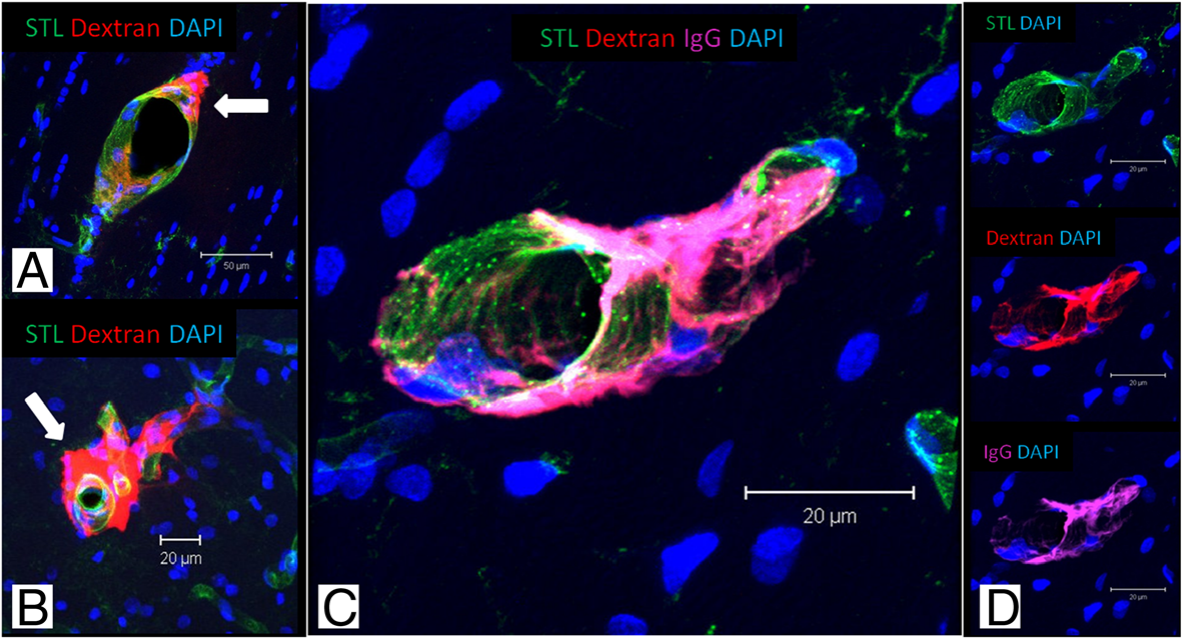
**Distribution of Dextran and demonstration of endothelial leaks.** Dextran (70 kDa) was applied in 30-32 weeks old SHRSP **(A-D)** under in vivo conditions and visualized by Rhodamin post mortem. **A** shows vessel wall adherent accumulations of Dextran (white arrow), in **B** perivascular deposits are detected (white arrow). Note the co-localization of IgG and Dextran in **C** indicating the detection of BBB breakdown by Dextran. **A** – corpus callosum, **B**, **C** – cortex; STL – solanum tuberosum lectin, IgG – Immunglobulin G, DAPI – 4′.6-Diamidin-2-phenylindol.

*In vivo* applied Dextran was labeled with Rhodamin and thereby visualized post mortem. Dextran could be detected in all investigated SHRSP and showed adherent accumulations to the vessel wall (Figure 5A, white arrow) and perivascular deposits (Figure 5B, white arrow). Dextran was co-localized with IgG (Figure 5C) indicating its capability for BBB breakdown detection.

## Discussion

Within the present pilot imaging study of SHRSP and Wistar controls we investigated the safety of 2 PM referring to the artificial surgical damage considered firstly against the background of small vessel vulnerability of SHRSP and secondly the sensitivity of this method to detect erythrocyte thrombi indicative of early CSVD stages. We demonstrated that artificial damage was comparable between both rat groups and mainly included the development of partial capillary thromboses and small bleeds. Indeed, most likely due to the short time span between the imaging procedure and the perfusion process with subsequent histological examination of the brains, no associated infarcts or large parenchymal hemorrhages developed. Contrary to our initial hypothesis, accordance between imaging of erythrocyte thrombi in 2 PM and confirmation of erythrocyte thrombi in HE was rather poor. Intravitally applied Dextran used for 2 PM small vessel visualization served as excellent marker for BBB breakdown detection. Further studies will focus on concurrent markers indicative of the endothelial dysfunction e.g. on the investigation of the accordance between intravital and postmortem Dextran accumulations associated with the vascular walls. We are currently performing intravital measurements of the cerebral blood flow (CBF) in small vessels, aiming to identify areas of hypoperfusion that may be associated with CSVD.

To the best of our knowledge, the nature and frequency of artificial surgical damage have not been reported in detail in mice or in rats following the removal of dura mater [21,23,25,26,30-32,38-41]. Thus, we cannot discuss our findings in relation to the data of other research groups. Indeed, artificial damage exclusively affected the small vasculature and resulted in insignificant fatalities. Thereby, dura mater removal with subsequent damage of cortical small vessels appeared to be the main source for the detected surgical injury. However, dura mater removal is implicitly necessary for imaging of parenchymal vessels in the rat brain [21,29,32,35,41] and thus, the hazard associated with the surgical damage is not related to the rat model used in experiments, with or without intrinsic vascular pathologies.

From our current point of view there are no convincing reasons that could provide a complete explanation of the low incidence of detection of erythrocyte thrombi with intravital imaging and histology. The possible causes could be attributed to the limitations of the methods employed. For example, the intravital definition of erythrocyte thrombi might not have been sufficient and the complete definition should include other phenomena; our chosen definitions for this study were in line with those of other groups [42]. Only few SHRSP and Wistar controls exhibited arteriolar erythrocyte thrombi, which should be more sensitive to detection with 2 PM compared to the erythrocyte thrombi in the capillary bed. As there is an age-dependent progressive spread of erythrocyte thrombi from the capillaries into the arterioles and small arteries, future studies should include older animals [12]. Moreover, extending the investigations to a higher number of animals and additionally increasing the 2 PM recordings per animal may also lead to a better agreement of imaging and histology data.

To our best knowledge, there is no current work providing details on parenchymal Dextran distribution in rats [21,23,25,30-32,41]. Here we showed for the first time that Dextran (70 kDa) accumulated within the walls of small vessels. It is therefore potentially comparable to Evans Blue [43-45] and thus could be used for in vivo detection of damage to the BBB. Compared to IgG, Fibrinogen and Fibronectin, the conventional markers of BBB breakdown, Dextran may yield more results by revealing even smaller endothelial leaks because of its comparably lower molecular weight (IgG 150 kDa [46], Fibrinogen 300 kDa [47], Fibronectin 440 kDa [48]). Lower molecular weight Dextrans (e.g. 4 kDa, 40 kDa) may be even more promising markers for minor endothelial injuries.

## Abbreviations

2 PM: Two-photon microscopy; AD: Alzheimer’s disease; BBB: Blood brain barrier; CBF: Cerebral blood flow; CSVD: Cerebral small vessel disease; DAPI: 4′.6-Diamidin-2-phenylindol; HE: Haematoxylin-Eosin; IgG: Immunglobulin G; MRI: Magnetic resonance imaging; PBS: Phosphate-buffered saline; PFA: Paraformaldehyde; SHR: Spontaneously hypertensive rats; SHRSP: Spontaneously hypertensive stroke-prone rats; STL-FITC: Solanum tuberosum lectin-fluorescin isothiocyanate; vWF: von-Willebrand-factor; WMH: White matter hyperintensities.

## Competing interests

The authors declare that they have no competing interests.

## Authors’ contributions

SN, CG and SSch directed the study, designed experiments, analyzed data and drafted the manuscript; SS was involved in establishing 2 PM; SS, CZB, SM, ROC and CK contributed to the manuscript writing and data analysis; HJH and KR funded major parts of the study and contributed to manuscript writing. All authors read and approved the final manuscript.
